# Rare Reconstruction of Four Metatarsals Using Kirschner Wires After a Severe Crushing Injury: Surgical Decision Making and Prognosis

**DOI:** 10.7759/cureus.49318

**Published:** 2023-11-23

**Authors:** Thomas H Suh, Henry Avetisian, Jacob Speechley, Jordan L Palmer, John D Bailey

**Affiliations:** 1 College of Medicine, Kirksville College of Osteopathic Medicine, Andrew Taylor Still University (A.T. Still University), Kirksville, USA; 2 College of Medicine, Jacobs School of Medicine and Biomedical Sciences at the University of Buffalo, Buffalo, USA; 3 Orthopedic Surgery, Northeast Regional Medical Center, Mid America Orthopedics & Spine, Kirksville, USA

**Keywords:** second metatarsal, open fracture reduction, kirschner wires, k-wire, type ii open fracture, foot trauma, fifth metatarsal fracture, open reduction fifth metatarsal fractures, third metatarsal, metatarsal bone fracture

## Abstract

Metatarsal fractures are one of the most common injuries after foot trauma. It is debilitating, as the metatarsals are one of the most crucial bones for any weight-bearing movement. This report demonstrates the beneficial outcome of using Kirschner wires (K-wires) in a trauma setting and the complicated healing process. A 56-year-old gentleman was brought into the emergency department after a reinforced cement pipe fell onto the patient’s steel-toe boots, striking his left foot immediately proximal to the steel portion of the boot. The patient had sustained displaced comminuted fractures of the left second, third, fourth, and fifth metatarsals with an extensive open wound (Gustilo type II open fracture). Open reduction with internal fixation (ORIF) was performed using K-wires to restore and preserve the anatomical and functional integrity of the foot. Following the surgery, the patient developed a hammer toe of the left fifth metatarsophalangeal (MTP) joint two months after the ORIF; we performed resection arthroplasty to relieve discomfort and further aid the recovery process. Following the resection arthroplasty, eschar had formed at the surgical site, extending from the lateral aspect of the left foot to the plantar surface, for which we had performed a skin graft after excisional debridement of the necrotic tissue. After one year of close follow-ups with rigorous physical therapy exercises, the patient had a fair recovery process and is now able to ambulate without any assistive devices. As such, using K-wires remains a viable option for reducing misaligned metatarsal fractures and providing fairly good outcomes even in the setting of severe foot trauma.

## Introduction

Metatarsalgia is defined as pain in the forefoot and can be related to disruption in the proper union of the metatarsals following trauma [1]. Therefore, it is crucial that proper alignment is obtained with proper techniques and appropriate hardware. Current guidelines suggest a firm-soled shoe or a walking boot along with soft dressing for a simple, non-displaced metatarsal shaft fracture; however, management of displaced comminuted metatarsal shaft fractures requires surgical intervention to preserve the structural and functional integrity of the bones [2,3]. It is known that retrograde percutaneous pinning using Kirschner wires (K-wires) preserves the vascularity of the bone with minimal disruption of the soft tissue. Furthermore, the usage of such wires promotes good visualization of the anatomy in case an invasive surgery should be performed for displacement reduction [1,3]. As such, K-wires provide a multitude of benefits, such as minimal dissection, early functional/anatomical recovery, and decreased chance of re-displacement [2]. 

## Case presentation

**Figure 1:**
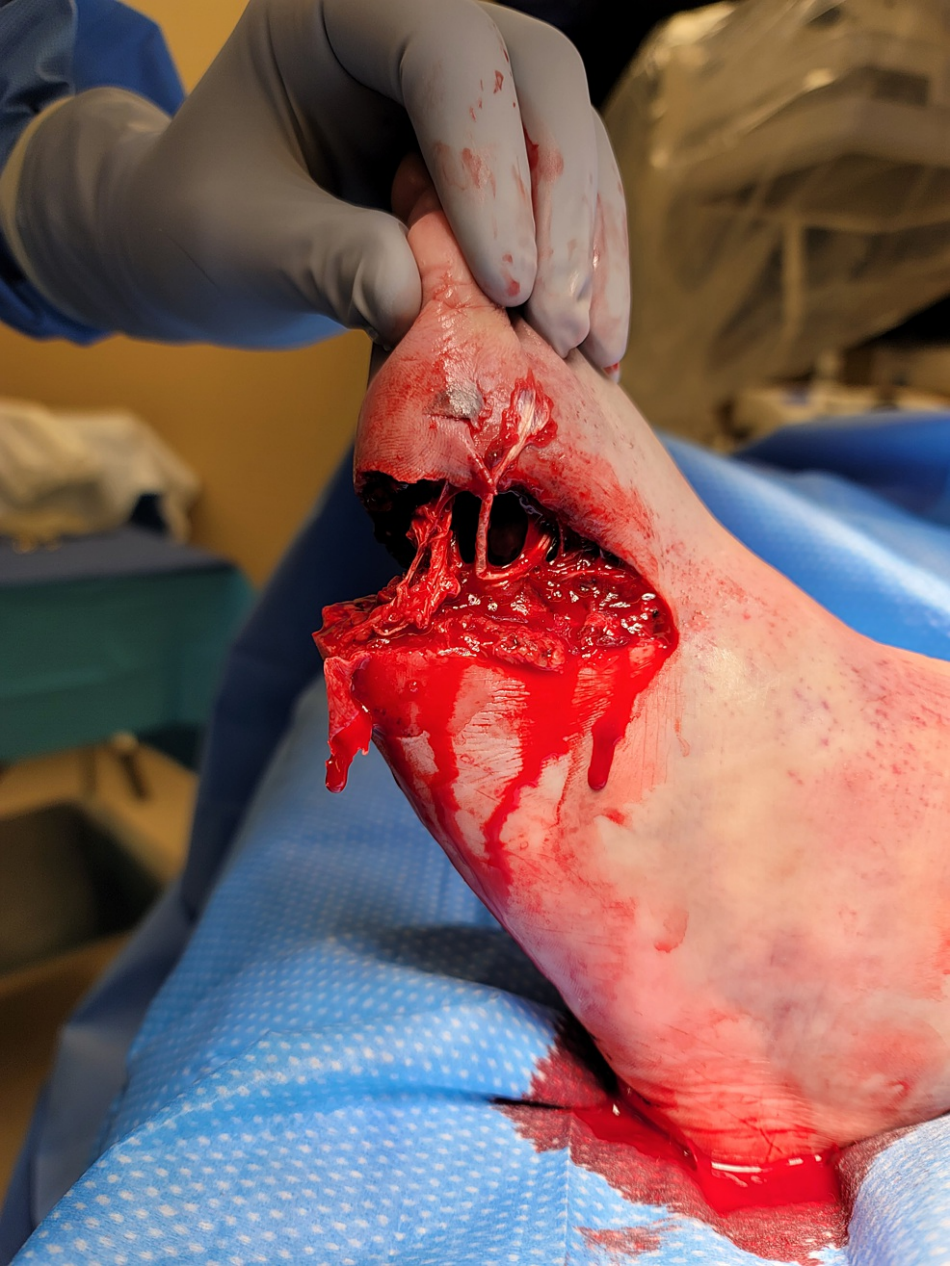
Gross image of the left foot wound Type II open fracture with open wound extending from the mid-dorsal aspect of the left foot, to the lateral aspect, then to the plantar surface in an oblique transverse fashion.

A 56-year-old gentleman with no pertinent medical comorbidities, surgical history, or social history was brought into the emergency department after a reinforced cement pipe fell onto the patient’s left steel-toe boot, striking his left foot immediately proximal to the steel portion of the boot. Upon physical examination, there was a Gustilo type II open fracture extending from the mid-dorsal aspect of the left foot to the lateral aspect, then to the plantar surface in an oblique transverse fashion (Figure 1). Palpable fractures and obvious deformities of the second, third, fourth, and fifth metatarsals were also noted. Further examination demonstrated complete anesthesia distal to the open wound, specifically in the fourth and fifth phalanges. Sensations were intact in the dorsal and plantar aspects of the left foot proximal to the open wound. Flexion and extension at the second, third, fourth, and fifth metatarsophalangeal (MTP) joints were absent, while the first MTP movements were minimally intact. In the ED, appropriate antibiotics, including cefazolin, and wound dressing were applied until the surgery was performed.

**Figure 2:**
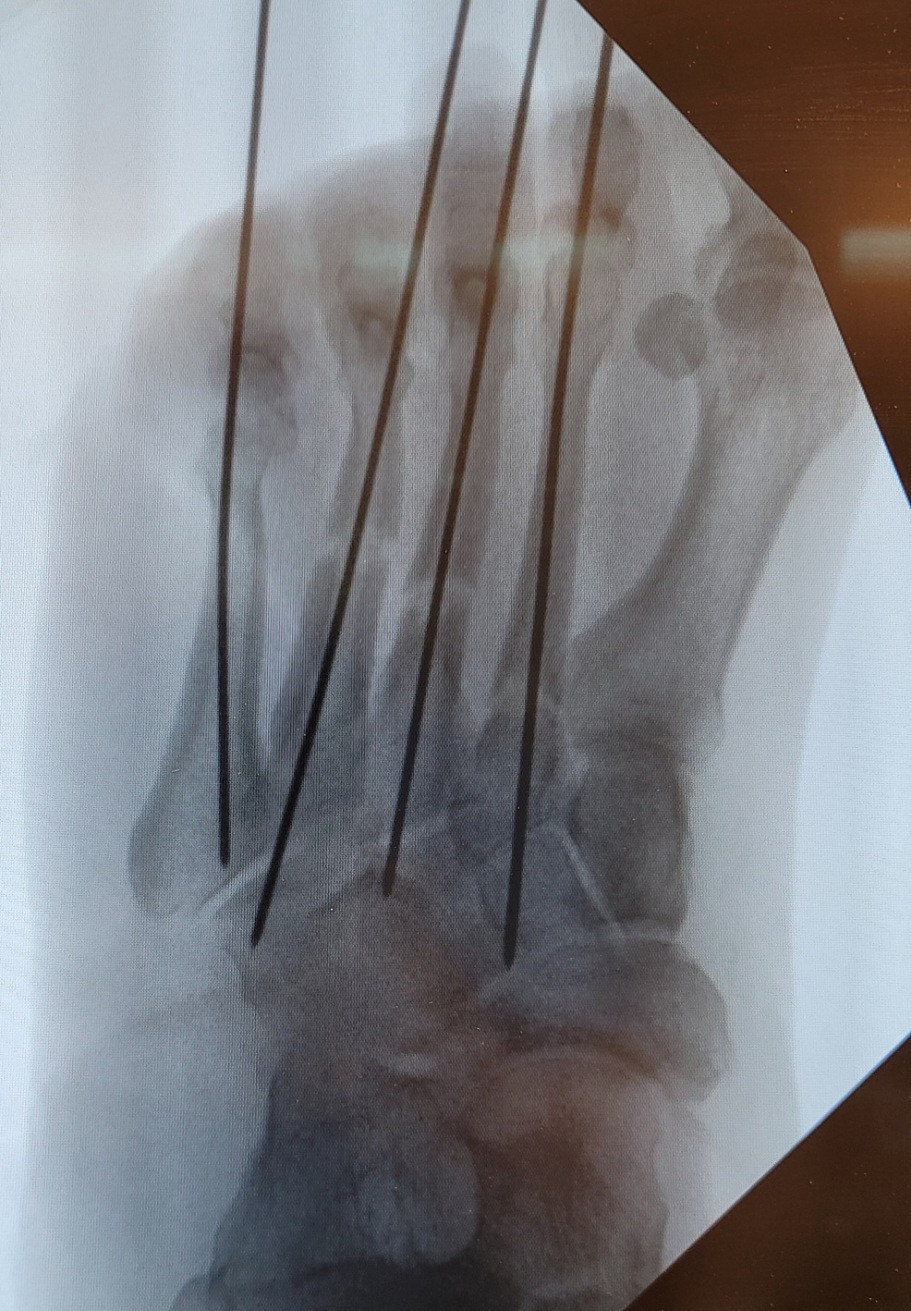
AP view of the left foot post-ORIF using K-wires (0.054-inch) AP view of the left foot shows apparent complete mid-shaft fractures of the second, third, fourth, and fifth metatarsals. K-wires (0.054-inch) are in place. ORIF: open reduction with internal fixation; K-wires: Kirschner wires

An X-ray of the left foot demonstrated displaced transverse fractures of the second, third, fourth, and fifth metatarsals (image not included). Flexors/extensor tendons were intact. Due to the nature of the fracture, percutaneous K-wire fixation was performed to the left second, third, fourth, and fifth metatarsals (0.054-inch) and excision debridement (Figure 2). The patient had an uneventful recovery with good wound healing following the operation and was discharged home three days later with a Penrose drain in place. With such extensive damage to the metatarsals, neurovasculature, and soft tissue, complete restoration was unachievable. During the recovery at home, the patient developed a hammer toe deformity of the fifth MTP joint two months after the initial injury due to the extent of the injury, for which we performed resection arthroplasty to relieve discomfort and prevent complications. The patient recovered well from the procedure. K-wires were removed eight weeks after the initial surgery.

**Figure 3:**
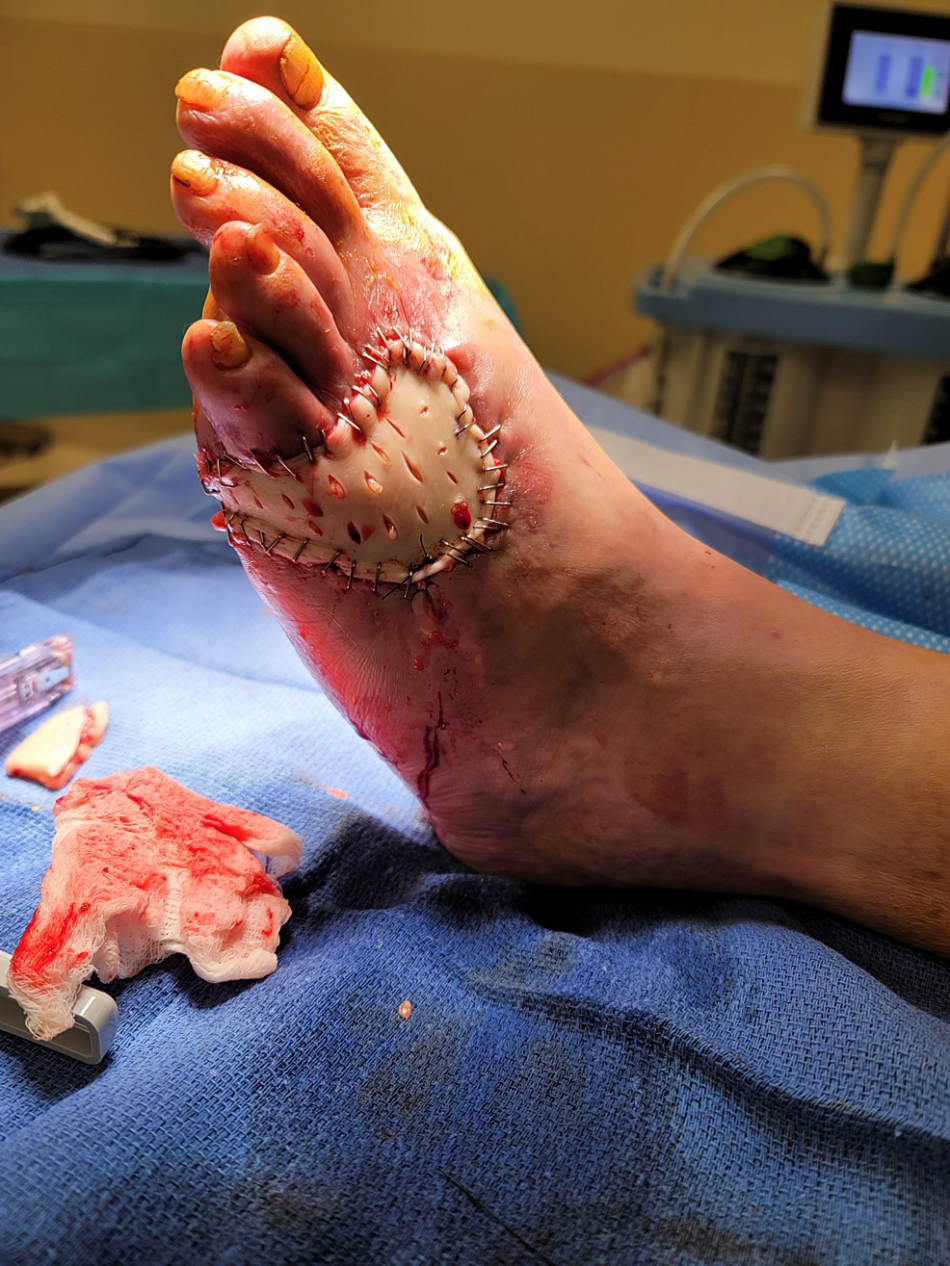
Left foot with skin graft in place Left foot with K-wires removed; skin graft in place after excisional debridement. K-wires: Kirschner wires

During the follow-up three months after the initial surgery, the patient was noted to have an eschar at the surgical wound with necrotic tissue. We performed excisional debridement of the necrotic tissue and a full-thickness skin graft from the left thigh (Figure 3).

**Figure 4:**
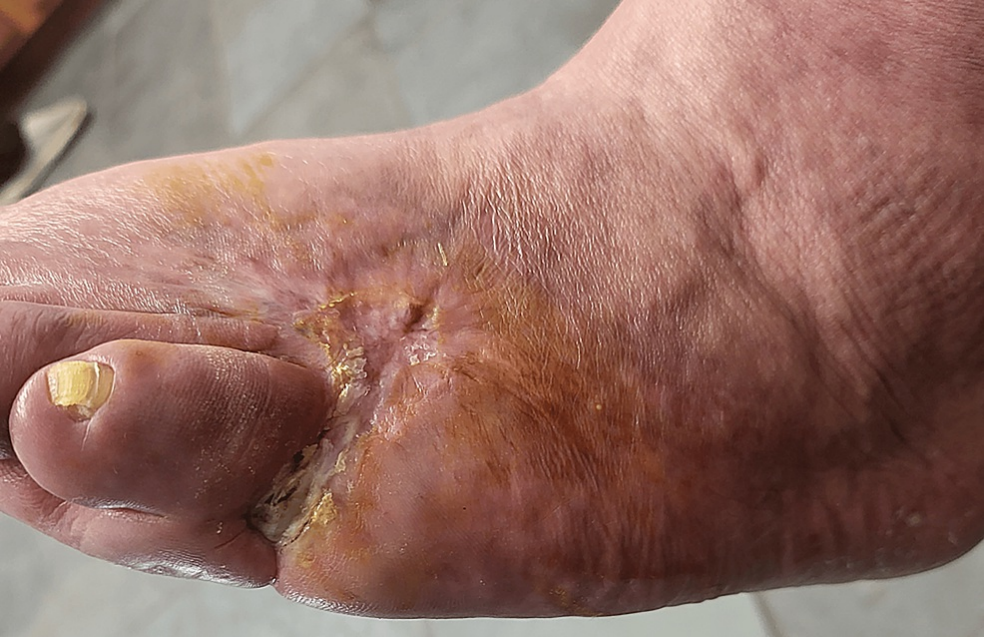
Left foot eight months after the initial injury During the follow-up eight months after the initial injury, the foot healed very well. No signs of acute infection were appreciated. The shortened left fifth toe is shown post-resection arthroplasty.

Following months of recovery after the initial injury, the patient was able to ambulate without any walking aid devices. Full functional recovery was achieved with the partial restoration of neurological integrity. Post-resection arthroplasty, the patient was able to flex and extend his left fifth toe, as well as the other remaining phalanges. The wound healed very well with no signs of infection (Figure 4). Sensation to the lateral portion of the left foot was partially intact with normal capillary refill times.

## Discussion

Although workplace injury involving the foot and ankle area commonly occurs, a case of multiple metatarsal fractures is relatively uncommon (16.7%) [4,5]. From this case, we have learned that although foot injuries have a relatively low mortality rate [6], it has a significant impact on the quality of life based on the EuroQol-5 dimensions (EQ-5D) score) [7]. Therefore, surgical fixation was necessary to optimize the reduction of bone misalignment and decrease the chance of postoperative complications [8]. By doing so, we sought to restore the normal function of the foot as well as baseline quality of life. 

After the percutaneous K-wire fixation, we were able to achieve optimal alignment and reduction of the displaced fractured metatarsals. From the follow-up, the patient was noted to have a good recovery process. By the eighth week post-ORIF, an X-ray of the left foot demonstrated a good union of the metatarsals (image not included). Sensations to the distal phalanges (distal to the open injury) were partially restored and vascular integrity had returned to baseline. Contrary to our beliefs, Samaila et al. demonstrated that the delayed healing process of the metatarsals is not necessarily due to the extent of the injury; rather it is more associated with patient factors, such as smoking, nutrition status, medical comorbidities, and immune status [9]. However, if the metatarsal injury involves multiple central metatarsal fractures, sagittal plane deformity >10 degrees, or >4mm translation, surgical intervention is indicated [10]. 

From this case, we seek to further promote literature on multiple metatarsal fractures in the orthopedics and trauma realm. At this time, limited information is available on the outcome of operative management of multiple displaced metatarsal fractures. Given the patient’s negative medical comorbidities, past surgical histories, or any pertinent social histories, this is a good example demonstrating the management of severe open fracture involving four metatarsals and its outcome.

## Conclusions

The case demonstrates the beneficial outcome of operative intervention in treating four displaced metatarsal fractures after such extensive workplace injury. Displaced fractures were well-aligned using 0.054-inch K-wires with proper closure of the wounds. Post-surgical complications included a hammer toe of the left fifth phalanx, which was addressed via resection arthroplasty, and a necrotic surgical wound, which was managed via skin graft with wound debridement. By week eight, K-wires were removed with direct visualization of the alignment of the metatarsals. A satisfactory result was achieved with a stepwise increase in function and quality of life.
